# A Microbial Monitoring System Demonstrated on the International Space Station Provides a Successful Platform for Detection of Targeted Microorganisms

**DOI:** 10.3390/life11060492

**Published:** 2021-05-27

**Authors:** Christina L. M. Khodadad, Cherie M. Oubre, Victoria A. Castro, Stephanie M. Flint, Monsi C. Roman, Charlie Mark Ott, Cory J. Spern, Mary E. Hummerick, Gretchen J. Maldonado Vazquez, Michele N. Birmele, Quinn Whitlock, Matt Scullion, Christina M. Flowers, Raymond M. Wheeler, Orlando Melendez

**Affiliations:** 1 Kennedy Space Center, Amentum Services, Inc., LASSO, Merritt Island, FL 32899, USA; cory.j.spern@nasa.gov (C.J.S.); mary.e.hummerick@nasa.gov (M.E.H.); 2 Johnson Space Center, National Aeronautics and Space Administration, Houston, TX 77058, USA; cherie.m.oubre@nasa.gov (C.M.O.); stephanie.m.flint@nasa.gov (S.M.F.); c.m.ott@nasa.gov (C.M.O.); 3 Johnson Space Center, Government Solutions, KBR, Houston, TX 77058, USA; victoria.a.castro@nasa.gov; 4 Marshall Space Flight Center, Science and Technology Office, NASA, Huntsville, AL 35808, USA; monsi.roman@nasa.gov; 5 Kennedy Space Center, Southeastern Universities Research Association (SURA), Merritt Island, FL 32899, USA; gretjmldo@gmail.com; 6 Kao USA, Cincinnati, OH 45214, USA; michele.birmele@kao.com; 7 BioFire Defense, Salt Lake City, UT 84107, USA; Quinn.Whitlock@BioFireDefense.com (Q.W.); Matt.Scullion@BioFireDefense.com (M.S.); Christina.Flowers@BioFireDefense.com (C.M.F.); 8 Kennedy Space Center, ISS Utilization, UB-A, NASA, Merritt Island, FL 32899, USA; raymond.m.wheeler@nasa.gov (R.M.W.); orlando.melendez-1@nasa.gov (O.M.)

**Keywords:** microbial monitoring, qPCR, International Space Station (ISS), microbiome, Environmental Control and Life Support Systems (ECLSS), closed environment, microgravity

## Abstract

Closed environments such as the International Space Station (ISS) and spacecraft for other planned interplanetary destinations require sustainable environmental control systems for manned spaceflight and habitation. These systems require monitoring for microbial contaminants and potential pathogens that could foul equipment or affect the health of the crew. Technological advances may help to facilitate this environmental monitoring, but many of the current advances do not function as expected in reduced gravity conditions. The microbial monitoring system (RAZOR^®^ EX) is a compact, semi-quantitative rugged PCR instrument that was successfully tested on the ISS using station potable water. After a series of technical demonstrations between ISS and ground laboratories, it was determined that the instruments functioned comparably and provided a sample to answer flow in approximately 1 hour without enrichment or sample manipulation. Post-flight, additional advancements were accomplished at Kennedy Space Center, Merritt Island, FL, USA, to expand the instrument’s detections of targeted microorganisms of concern such as water, food-borne, and surface microbes including *Salmonella enterica* serovar Typhimurium, *Pseudomonas aeruginosa*, *Escherichia coli,* and *Aeromonas hydrophilia*. Early detection of contaminants and bio-fouling microbes will increase crew safety and the ability to make appropriate operational decisions to minimize exposure to these contaminants.

## 1. Introduction

The International Space Station (ISS) is a closed environment that must maintain safe and sustainable air and water systems. As the ISS supports rotating crews and additional spacecraft, including new hardware to be installed, it is necessary to monitor the microbial flora as there are new introductions of microorganisms with each crew and cargo rotation [1,2]. Air, water, surfaces, Environmental Control and Life Support Systems (ECLSS), and other flight hardware are at risk without having the capability of monitoring the abundance and richness associated with microbial assemblages [3]. Current ISS microbial monitoring methods used to detect and minimize contamination are laborious and time-intensive and provide only a limited capability to enumerate bacterial or fungal cells [4,5,6]. Because current methods cannot characterize or identify the organisms needed for microbial characterization on orbit, samples are returned to ground for further microbial characterization and identification. This delays any necessary remediation, placing the crew and equipment aboard the ISS at risk from potential pathogenic or biofouling microbes. Though this may be adequate at this time, it will not be sufficient for future long-duration lunar or Martian manned space flight missions which will have limited ground access.

**Figure 1:**
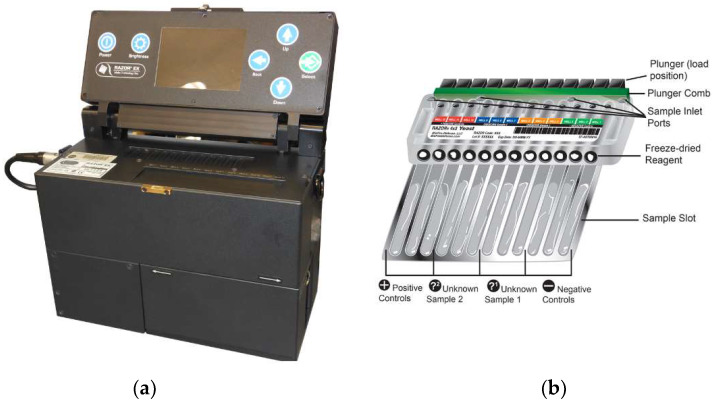
The RAZOR^®^ EX (**a**) and a 4 × 3 configured pouch (**b**). (Courtesy of BioFire Defense, LLC, Salt Lake City, UT, USA.)

An inter-agency meeting was held at NASA’s Johnson Space Center (JSC), Houston, TX, USA, in 2011–2012 to investigate alternatives to current microbial monitoring methods that were available and the feasibility of each functioning in microgravity as well as for future long-duration space flight missions. The team unanimously recommended polymerase chain reaction (PCR) as the most comprehensive and feasible microbial monitoring methodology currently available for that time [7,8]. Subsequent to this decision, investigation into numerous PCR instruments commenced and after testing and an in-depth trade study, the RAZOR^®^ EX, BioFire Defense, LLC (Salt Lake City, UT, USA) was selected for a technical demonstration aboard the ISS as part of the Water Monitoring Suite, (WMS) 2015 (Figure 1) [1,2,9].

The RAZOR^®^ EX is a field-portable, real-time, semi-quantitative PCR instrument designed for use by the military and first responders for the detection and identification of microorganisms in environmental samples. Its attributes were deemed ideal for spaceflight testing based on criteria and decisional outcomes at a NASA workshop as well as subsequent engineering approaches and it was considered to be a commercial off-the-shelf unit (COTS) (Table 1) [2,7,8,9].

Current microbial monitoring aboard the ISS uses non-specific culturing methods that can neither accurately enumerate nor identify microorganisms present in the environment [4,5,6]. Generally, only 1% of the microorganisms present in the environment can be successfully cultured and results can be misleading as to the presence or absence of microorganisms, including those pathogenic to humans [10,11,12]. The very process of culturing cells proliferates the potential number of microorganisms, creating unsafe concentrations of microbes and increasing the level of risk to crew health.

PCR is a molecular-based technique that can be used to rapidly and accurately identify targeted microorganisms. The broad library of organisms that can be identified include those that are potential human pathogens found within the environment aboard the ISS. These microorganisms can be detected at a low level of detection (LLOD) without the need to enrich or concentrate the samples [1,6,13,14]. Real-time PCR capabilities on station would allow for accurate identification of single or multiple targeted microorganisms simultaneously, reducing or eliminating a dependence on-ground sample analysis and thus allowing for rapid mitigation. Implementing a rapid detection method would radically improve the current ISS microbial monitoring capacity while decreasing the exposure of crew members to potential pathogens.

Current research on spacecraft environments shows that microorganisms are continually transferred from crew to vehicle subsystems including environmental control and life support systems (ECLSS), and potentially back to the crew [15,16]. Studies completed on ISS have identified numerous taxa present throughout the ISS in both air and surface sampling [15,16,17]. Microorganisms can have direct impacts on crew health, the function of vehicle systems, and the safety of food production systems. Within the ISS, ECLSS not only maintains an environment suitable for sustaining a human crew but also unintentionally selects for a persistent, human-associated microbiome. In closed environments such as spacecraft, the relative abundance of human-associated microbes closely related to human pathogens may increase with mission duration. Capabilities to analyze samples on orbit are limited or not yet validated, and therefore biotechnological advances are needed to ensure our goals of safe spaceflight to the moon and Mars [18].

In fiscal year (FY) 2016, the RAZOR^®^ EX tested at Kennedy Space Center, Merritt Island, FL, USA (KSC), as a microbial monitoring platform was launched to ISS aboard SpaceX-10. The RAZOR^®^ EX system, a semi-quantitative PCR instrument that amplifies DNA to identify target organisms, was tested in microgravity using ISS water resources obtained from the potable water system. The goal of the testing was to validate the consumables and chemistry associated with the PCR reaction and station potable water dispenser, the hardware, and procedures for flight operations for crew members without prior exposure or instructions on the instrument. The completion of these demonstrations led to additional advancements on the ground at KSC to further the development of additional assays with a direct application to food safety and to determine the longevity of the materials and PCR reagents. Rapid detection of select microbes could help ensure the health and safety of the crew as well as to help characterize the ISS microbiome.

## 2. Materials and Methods

### 2.1. Preflight Preparations

Initial hardware functional tests were performed using the RAZOR^®^ EX flight and engineering hardware units to validate the hardware’s functionality, pouch chemistry and testing methods prior to flight. The flight 4 × 3 pouches (Figure 1) prepared at BioFire Defense, Salt Lake City, UT, USA, contained lyophilized PCR reagents, gene-specific primers, TaqMan probes, and *Pseudomonas aeruginosa* ATCC 10145 DNA with detection level requirements for 10^2^ to 10^6^ cells/mL equivalents based on genome size. The 4 × 3 configuration allows for four different samples to be completed in triplicate. In this case, the wells were prepared to test three samples and a no template control. DNA was isolated from pure culture and purified at KSC using the Microbial DNA Isolation Kit (Qiagen, Inc., Carlsbad, CA, USA) per vendor protocols. DNA quality was confirmed with Nanodrop 260/280 ratio then provided to the vendor in TE buffer for incorporation into the pouches. PCR reagents and templates were prepared in optimal concentrations, pre-loaded into RAZOR pouches, and lyophilized for stability until reactions could be processed. Each well contained 1X reaction buffer, 1.5 mM MgCl_2_, 200 µM each dNTPs, 500 nM each primer, 250 nM TaqMan probe, and the polymerase enzyme. Primers for *gyrB* gene of *P. aeruginosa* (5′-3′F-GGCGTGGGTGTGGAAGTC and R-TGGTGAAGCAGAGCAGGTTCT) were previously described by Lee, et al. (2011) along with the TaqMan probe (TGCAGTGGAACGACA) (Table 2 and Table 3). 

Pouches were created in two separate 4 × 3 configurations and contained all required PCR reagents including the DNA isolated from *P. aeruginosa* (Table 3). The variable was the concentration of the DNA template added to each well. The low concentration pouch contained template DNA in 0.2 ng, 0.02 ng, and 0.002 ng, while the high concentration pouch contained DNA concentrations of 0.2 ng, 2 ng, and 20 ng. The 0.2 ng DNA samples were contained in each pouch as an internal indicator of consistency and as a bridge to samples run consecutively in time. Each concentration was run in triplicate and a no template control was run simultaneously within each pouch. This theoretically corresponded to 10^6^, 10^5^, and 10^4^ cells per reaction for the high concentration pouch and for the low pouch it corresponded to 10^4^, 10^3^, and 10^2^ cells per reaction (based upon the genome size for *P. aeruginosa*). The testing performed used three water samples also planned for flight operations which included molecular grade water, archived water from the ISS, and 0.2 µm filtered archived water from the ISS. A 1 mL aliquot of each water sample was drawn into a 3 mL easy glide syringe fitted with a cannula. The cannula was inserted into the wells via the sample insert port and a sufficient aliquot to fill each chamber (300 µL) was drawn automatically into each chamber. Each syringe was used to fill 2 chambers for a total of 600 µL. This left approximately 300–400 µL residual in the syringe, but also reduced disposable up-mass. PCR conditions included 94 °C for 4 min, followed by 94 °C for 30 s, and 60 °C for 90 s for 45 cycles (Table 4). The critical threshold level or crossing point (Cp) was recorded, and data obtained from each instrument and PCR results were compared. This testing also completed end-to-end science verification validating the testing methods and procedures.

### 2.2. Flight Testing

The RAZOR^®^ EX instrument and prepared PCR pouches were launched to the ISS aboard SpaceX-10 in July 2016. Ground and inflight testing were performed between September 2016 and April 2017. Ground controls were completed at both KSC, FL, USA, and JSC, TX, USA, on two separate instruments with a 24 h delay from the ISS test procedure. PCR reagents and templates were prepared in optimal concentrations as described in preflight testing. Testing was performed using 3 different water samples for flight operations and ground controls including molecular grade water, water from the ISS potable water dispenser (PWD), and filtered (0.2 µm) ISS water, also from the PWD. PCR conditions were the same as described in preflight validation. The critical threshold level (Cp) was recorded and data obtained from both flight and ground PCR reactions were compared for similarities or differences using a *t*-test (*p* < 0.05). Samples were then pooled.

### 2.3. Post-Flight Advances

Upon completion of the PCR reactions with RAZOR^®^ EX microbial monitoring system flight data, additional detection assays were developed and validated at KSC for targeted microorganisms including *Escherichia coli*, *Salmonella enterica* serovar Typhimurium (hereafter termed *S*. Typhimurium), *Aeromonas hydrophilia*, and an assay to quantify total microbial load using a 16S rRNA gene which could detect up to 98–99% of bacterial species. (Table 2). The PCR assays (primers and probes) were developed using specific genes that identify the species taxonomic level. The genes were identified through the scientific literature and then further evaluated for specificity to the taxa selected (Table 2). Each set of primers and probes were confirmed for their specificity using PCR for the targeted and numerous non-targeted bacteria. Confirmation was completed on the Roche Lightcycler 480 and the RAZOR^®^ EX. All primers and probe combinations were acquired through Invitrogen, Inc. (Grand Island, NY, USA).

### 2.4. RAZOR^®^ EX Pouch Longevity Study

RAZOR^®^ EX pouches and lyophilized pouch reagents were produced by BioFire Defense, (Salt Lake City, UT, USA), placed under vacuum, packaged, and assigned an expiration date of 12 months post-manufacture. Pouches containing lyophilized reagents were tested for quality control upon receipt establishing a baseline for comparison to results obtained at later dates. To determine if the storage of pouches and the longevity of the lyophilized PCR reagents would exceed the estimated shelf life, pouches were tested monthly on ground for comparison of performance over time. Pouches that contained all required PCR reagents and DNA, similar to inflight and ground tests, were packaged in 2015 and expired in 2016. Pouches were visually inspected for the vacuum seal as the vacuum is critical to pouch and reagent performance. Runs were completed monthly and compared to the first ground test of these pouches completed in 2015. Differences were noted regarding the critical threshold values for each concentration. Empty reagent pouches were also maintained and tested over time using liquid reagents to determine the efficacy of the plastic materials and vacuum used in the assembly of these pouches. Empty pouches being tested were manufactured in 2013 and expired in 2014. Testing continued into 2019.

### 2.5. Application for Food Aampling

Two methods of sampling (swab and adhesive tape) were conducted to recover a known concentration of microbes from tomato fruit and quantified by heterotrophic plate counts and RAZOR^®^ EX pouches with the optimized *Salmonella* assays. *S.* Typhimurium was sub-cultured from frozen stocks and maintained on trypticase soy agar plates (TSA) with weekly transfer to fresh plates. For testing, a single colony was cultured in 25 mL trypticase soy broth (TSB) at 30–37 °C for 15 to 18 h at 125 rpm in a rotary shaker. Cultures were centrifuged at 5000 rpm for 10 min, washed in phosphate buffered saline (PBS), and ultimately re-suspended in sterile water. Cell concentration was determined using the GENESYS Spectrophotometer (Thermo Fisher, Waltham, MA, USA) (0.1 abs at 540 nm = 10^8^ cells) and serial dilutions to the desired concentrations were completed and plated on TSA agar to confirm concentrations (CFU/mL). Plates were incubated at 37 °C for 24 to 48 h.

A 50 µL aliquot of *S.* Typhimurium was placed by approximately 5–10 drops onto a 1 cm area of Red Robin tomato (*Solanum lycopersicum* cv RRT) of cell densities of 10^6^, 10^7^, and 10^8^ and allowed to dry for approximately 3 h in a biological safety cabinet (BSC). Each were completed in triplicate and then aseptically sampled with tape (3M, St. Paul, MN, USA), swab, or manually shaken in a Labco bag (fruit only), which served as a control to determine the recovery rate. Water served as a no template control. One swab wetted with sterile water sampled an area covering 1 cm^2^ with a horizontal then vertical back and forth motion. Tape (19 mm × 50 mm) was pressed onto the fruit with the sample aliquots and peeled back, removing the cells. Each swab or tape was placed into a small vial containing 5 mL of sterile water and shaken vigorously by hand for 30 s. Each sample was also injected into prepared RAZOR^®^ EX pouches with the species-specific assays and compared to a standard curve for recovery concentrations. The *invA* gene specific to *S.* Typhimurium are found in one copy per cell, therefore, it can be directly related to concentration of the recovered cells.

A standard curve was generated using a serial dilution of cell culture from 10^2^ to 10^6^ cells/mL and used to determine sample concentrations (R^2^ = 0.995). Values were compared using the Student t-test and standard deviation was calculated in Microsoft Excel or PRISM. Determination of differences between counts (log transformed CFU) (*p* < 0.05) were performed using a two-way ANOVA followed by Tukey’s multiple comparison test in GraphPad (GraphPad Prism version 8.0.0 for Windows. GraphPad Software, San Diego, CA, USA, www.graphpad.com).

## 3. Results

### 3.1. Preflight

**Figure 2:**
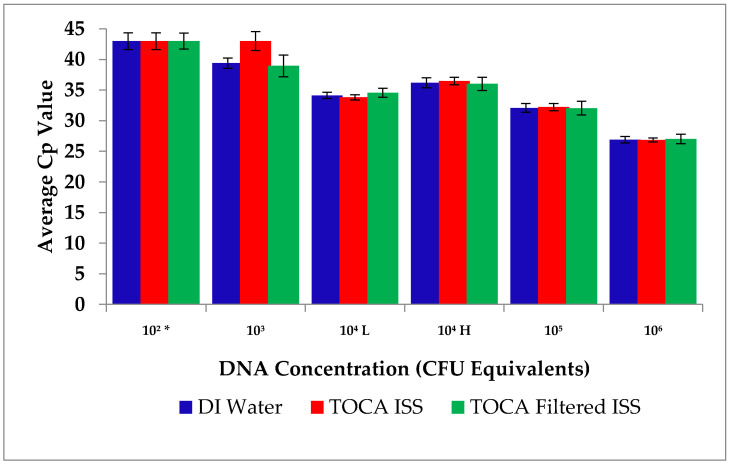
Science Verification Test (SVT) results for the Microbial Monitoring System (RAZOR^®^ EX) completed at JSC, March 2016 prior to flight. Average Cp for each water type (n = 3 pouches). Archived ISS water was acquired from the total organic carbon analysis (TOCA) sampling taken monthly on ISS. The (*) Cp for all 1 × 10^2^ wells were extrapolated as it occurred in the last five cycles of the PCR run. (L) indicates the low concentration pouch and (H) indicates the higher concentration pouch. Error bars are standard deviation.

A series of pouches were tested on both the flight and ground instruments with archived ISS and molecular grade water at JSC during a science verification test (SVT). These tests validated the payload including the pouch configuration for flight (Figure 2).

All DNA samples (*P. aeruginosa*) were detected as expected. Data were pooled to determine average Cp (Figure 2). DNA amplification doubling time was between 3 and 4 cycles with each dilution, and the pouch chemistry was successfully detected with all water types (Figure 2).

### 3.2. Flight Samples

**Figure 3:**
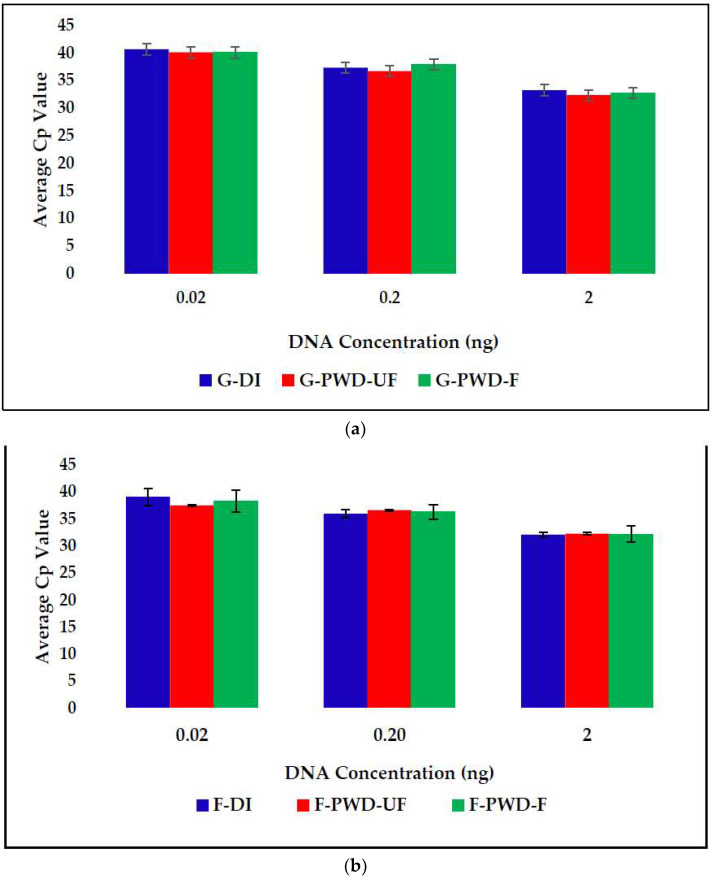
A comparison of ground tests (**a**) versus flight tests (**b**) for 3 concentrations of *P. aeruginosa* DNA, with DI water, potable water dispenser (PWD), and unfiltered (UF) and filtered (F) water. Nanograms of DNA are equivalent to 10^2^, 10^3^, and 10^4^ CFU equivalents based on genome size. Error bars are standard deviation (SD).

Three water sample types were also tested in flight beginning in September 2016 (Figure 3). Each ground and flight instrument performed as expected, successfully amplifying the *P. aeruginosa* DNA with each water type without chemical interference to the PCR reactions. Ground samples were completed within 24 h of the flight sample processing; there appeared to be no significant difference in Cp values between flight and ground samples or between flight and ground RAZOR^®^ EX instruments (Figure 3).

### 3.3. Longevity Study

**Figure 4:**
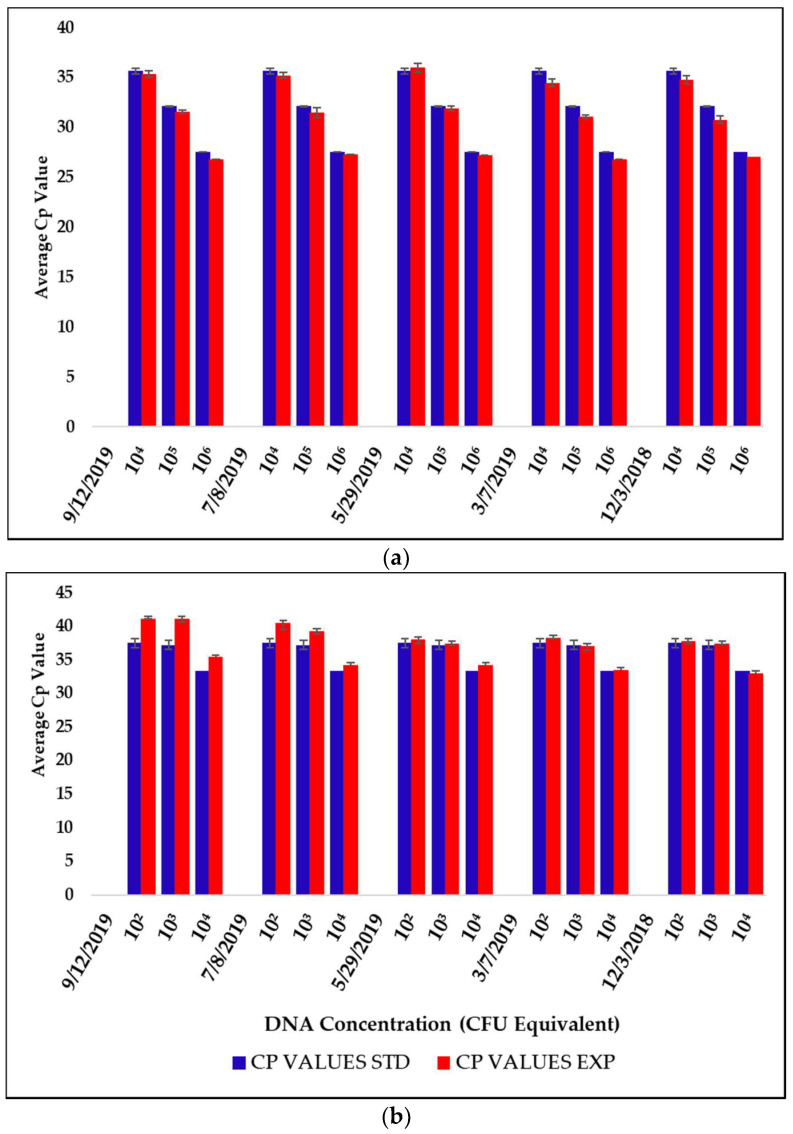
Graphs of the average threshold Cp for the longevity study including 5 runs each of *P. aeruginosa* high concentration (**a**) and *P. aeruginosa* lower concentration (**b**) RAZOR^®^ EX pouches. Pouches were manufactured in 2015, expiring in 2016. Error bars are standard deviation (SD).

Reagents and pouches for the microbial monitor have a shelf life of 1 year guaranteed per vendor specifications (BioFire Defense, LLC, Salt Lake City, UT, USA). This would be insufficient time for long-distance/long-duration spaceflight missions, and it required further investigation into the longevity of reagents and pouch materials (plastic). Empty pouches that were produced in 2013 were used in the laboratory with liquid reagents to determine the longevity of the pouch material and the reagent pouches were produced in January 2016. Empty pouches were tested in the lab with established assays and compared to assays performed during their valid shelf life time frame. There was no detection in the loss of the vacuum seal on any pouch through 2019. Pouch wells began to show indication of material degradation in 2019 when the liquid reagents would not transfer into the lower pouch due to fused plastic layers. These pouches, which expired in 2014, were 5 years past the expiration. Pouch reagents produced in 2016 were monitored through 2019 and compared to values acquired upon receipt of the pouches in 2016. Higher concentration pouches appeared to show no loss of integrity; however, those pouches with lower concentrations of control DNA appeared to show indications of variability in the Cp values, indicating that a level of degradation was occurring (Figure 4). All controls remained negative with no amplification detected past the 45 cycles. All control wells remained negative with no amplification indicated out to 45 cycles.

### 3.4. Application for Food Sampling

**Figure 5:**
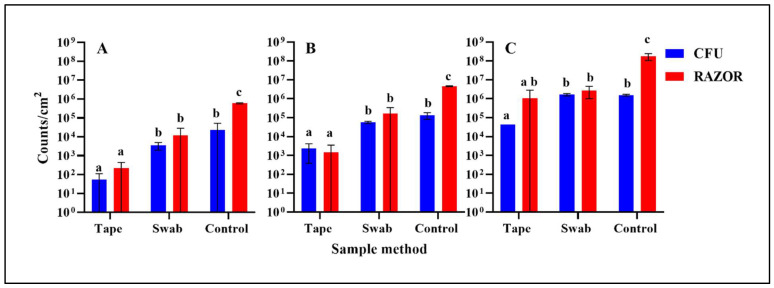
*S.* Typhimurium cell counts per cm^2^ on inoculated tomato surface determined by RAZOR^®^ EX PCR (red) and CFU plate counts (blue). Three inoculation cell densities were tested: A = 10^6^, B = 10^7^, C = 10^8^. Three sample methods were compared. Mean and individual values are shown in bars. Different letters indicate significant differences (*p* < 0.05) between sample method and detection method.

The Microbial Monitor RAZOR^®^ EX detection of microbes in solution was compared to the number of recovered cells from heterotrophic plate counts (HPC) using known concentrations of *Salmonella* inoculated onto a square centimeter surface of tomato (Figure 5). Cells were recovered with tape or swab and suspended in water. To determine the number of recovered *Salmonella* cells, the critical threshold levels for each sample were compared to a standard curve created with known concentrations of cells (Figure 5). The *invA* gene of *S.* Typhimurium is present in one copy per cell and therefore may be equated to the number of copies present. This value was then compared to the HPC (Figure 5). The accuracy of the comparison was determined by the slope of the standard curve.

The values of the samples were compared to the CFU values obtained from selective media plate counts, which indicated that RAZOR^®^ EX was able to detect microbes in each sampling method (Figure 5). For each concentration, RAZOR^®^ EX detected as well as or better than both the controls and the swab sampling methods. However, the tape sampling method recovery was lower than the recovery seen in the plate counts.

Two additional assays, for *Aeromonas hydrophilia* and the 16S rRNA gene for total microbial load, were developed and optimized in the lab at KSC for future use in detection of the microorganisms.

## 4. Discussion

Testing of the Microbial Monitoring System in flight confirmed that the system (RAZOR^®^ EX) was effective in microgravity and had high reproducibility and accuracy. As a PCR instrument, the RAZOR^®^ EX operated by three different astronauts on ISS during the testing period was easy to use and provided sample to answer, real-time information within approximately 1 h. In a case of a suspected anomaly, this could provide the crew with the ability to make rapid, operational decisions as the RAZOR^®^ EX and pouches require little to no sample preparation (i.e., no DNA isolation is required) because raw liquid sample may be directly inserted into the pouch containing the PCR reagents. With no sample preparation as required with many other instruments, the RAZOR^®^ EX simplified the process, which could save crew man-hours. In addition, ground testing of assays developed at KSC and by the vendor has provided evidence that the RAZOR^®^ EX could identify organisms to the species taxonomic level.

Not all microorganisms have a detrimental effect. A determination of targeted organisms of concern would be an important predetermination for this MMS/RAZOR^®^ EX to be a useful asset. First responders in the US government and local communities successfully utilized this instrument on Earth to provide information for safety or exposure of the investigating participants. It was also successfully used for water and food testing for select microorganisms in remote areas as well as for hazmat (https://www.biofiredefense.com/products/razorex/11/09/2020 (accessed on 11 September 2020)) (BioFire Defense, LLC; personal communication). This, however, requires prior knowledge of the threat and planning in order to have an appropriate assay for the detection of the organism. Other than the assay capable of determining total microbial load, each assay would have to be pre-determined and validated beforehand.

On ISS, water from the potable water dispenser (PWD) passed through a series of filtrations and chemical treatments and was tested in a filtered and unfiltered state. Filtration was completed to also determine whether chemical additives (salts, thiocyanates) in the water collection bags would have a negative effect on the PCR chemistry. PCR chemistry can be influenced by multiple classes of inhibitors [19], which may have a negative effect by decreasing reaction efficiency or totally inhibiting the reaction. There appeared to be no negative influence in the PCR reactions due to any chemical interference as both ground and flight samples performed similarly and there was no difference between water sample types.

To date, PCR reagents appear stable and produce results like those obtained at first testing in early 2016, as indicated in Figure 4. These data would indicate that the shelf life of PCR reagents could extend past the guaranteed shelf life by several years. This would be an important criterion to consider for long-duration missions or long-term storage in remote areas. However, we currently have not tested the shelf life of these materials and reagents under spaceflight conditions where environmental factors such as microgravity and radiation may have an effect. This determination would require additional time-course studies.

The microbial monitor provided a clear, visual answer to the user in a short time frame (approximately 1 h) with little to no data analysis required by crew members. At the completion of each PCR run, there are three visual outcomes immediately presented to the user, eliminating the usual logistic curve analyses. A logistic curve is presented on the screen along with a positive/negative (+/−) color-coded presence/absence indicator. Using the onboard wizard, an additional description for each well is provided clearly stating detection (or absence) for each pouch well. Again, this eliminates any questions of microbial detection, enabling faster decisions and mitigation, if required. The in-flight performance has reinforced the microbial monitor’s ability in spaceflight application to accurately detect and display the results of the PCR reactions.

Not only is it important to have an instrument that operates properly, but the materials and reagents are vital to success, especially during long-duration flights that surpass the shelf life of these peripherals. Long-duration missions will not have the benefit of resupply from Earth, and therefore long shelf life with minimal storage requirements is important. On Earth, most PCR reagents do have a shelf life of a year or less, if stored at required temperatures such as −20 °C to maintain integrity. The reagents utilized with the Microbial Monitor/RAZOR^®^ EX PCR instrument are lyophilized and maintained under vacuum in a sealed pouch with a 1-year manufacturer’s shelf life stored at ambient temperature. This study of the pouch components stored for several years at ambient temperatures yielded a favorable, longer shelf life (3 years for reagents and 6 years for pouch materials), allowing additional time for adequate, projected re-supply to the lunar surface or Mars.

Long-duration missions will require the need to maintain a fresh food supply as resupply will be infrequent. Crops in the form of leafy greens are grown on ISS to supplement astronauts’ diet and samples are returned to Earth for microbial analysis [20,21]. Without the ability to rely on Earth resources, the crew will need a reliable monitoring system to verify a safe food source. To approach this, two methods (swabs and tape) were used in the lab to determine a potential sampling method and investigate bacterial detection of food borne microbes such as *S.* Typhimurium. These sampling methods have been tested in the laboratory at KSC for sampling various crops and swabs are used on the ISS for sampling the Veggie hardware. *S.* Typhimurium is a microbe of concern as it can persist on food and cause extreme illness in humans who consume contaminated food. NASA has a zero-tolerance/acceptance for *S.* Typhimurium. Early data acquired in our study indicated that the microbial monitor could indeed detect various levels of *S.* Typhimurium from tomato fruit. As a proof of concept, this study was conducted at a high level of detection, but in previous lab tests this assay has detected 10^2^ cells/mL or lower [1]. In addition, the sampling method could influence the level of detection. We sampled the fruit with adhesive tape and swab sampling methods that could easily be used and processed by astronauts in microgravity to determine food safety. The swab sample recovered more of the microbes than the alternative tape sampling method. This may be due to the mixing procedure, in that the microbes may have re-adhered to the tape and were insufficiently mixed just prior to analysis. Though preliminary, these data provide a step forward for monitoring the fresh crops grown on the ISS.

We have presented and discussed the attributes and performance of a microbial monitor tested on ISS for the PWD and tested the system on Earth to expand its capabilities. The monitoring system improves the capability of detection above what is currently achieved on ISS and can provide rapid and reliable on-orbit detection and identification of up to 10 biological pathogens or bio-fouling microbes, simultaneously. Assays developed at the vendor and at KSC laboratories include other microbes such as *E. coli*, *A. hydrophilia*, *P. aeruginosa*, and total bacterial load using the 16S rRNA gene. Without having a requirement for enrichment, it could reduce the potential health risk to crew members. However, it is important to note that the MMS/RAZOR^®^ EX is a PCR instrument that will identify and quantify only targeted organisms selected and will not provide a community level analysis, such as that achieved with sequencing as seen with Oxford Nanopore Minion studies on ISS.

Having a cutting-edge instrument available for microbial monitoring with the required longevity of reagents is vital to mission success and will be a critical consideration for long-duration missions where microbial contamination might increase in a continually closed environment.

## Figures and Tables

**Table 1:** Microbial Monitoring System (RAZOR^®^ EX) attributes.

RAZOR EX^®^ Instrument Attributes	
Number of samples	12 total wells
Volume of sample per well	100 µL
Size (cm) (W × D × H)	25.4 × 11.4 × 19
Weight (kg)	4.9
Power	24 V power supply
Reagents	Lyophilized and pre-loaded pouch
Time to answer	30 min to 1 h
Sample type	Unprocessed sample, DNA or RNA
Universal 16S rRNA gene detection	LLOD 1 × 10^2^ copies

**Table 2:** PCR detection assays for targeted microorganisms, selected genes, function, and amplicon sizes in base pairs (bp). Reference for each primer and/or probe is noted.

Target Microorganism	Gene	Gene Function	Amplicon (bp)	Reference
*Escherichia coli*	*uidA*	Glucuronidase	82	Frahm, E. and Obst, U., 2003
*Salmonella enterica* serovar Typhimurium	*invA*	Invasion protein gene	119	Hoorfar et al. 2000.
*Pseudomonas aeruginosa*	*gyrB*	gyrase subunit B	67	Lee et al., 2011
*Aeromonas hydrophilia*	*aha1*	major adhesion protein	60	Lee et al., 2006
Universal Bacterial Primers	16S	ribosomal RNA gene	123	Suzuki et al., 2000.

**Table 3:** Final optimized PCR chemistry for various genes in a final volume of 150 µL. All reagents were obtained from BioFire Defense, Inc.^TM^ for quality of final optimization prior to lyophilization.

Reagent	Starting Concentration	*E. coli*	*Salmonella*	*Aeromonas*	16S
Stabilization buffer	4X	1X	1X	1X	1X
MgCl_2_ with BSA	Variable			3.0 mM	
2.5 mM each dNTP’s	25 mM	200 nM	200 nM	200 nM	200 nM
TaqStart Antibody	5 µg/µL	0.4	0.4	0.4	0.4
Forward Primer	100 µM	900 nM	100 nM	1000 nM	500 nM
Reverse Primer	100 µM	900 nM	100 nM	1000 nM	500 nM
TaqMan Probe	100 µM	200 nM	100 nM	250 nM	100 nM
Vtaq DNA polymerase Glycerol Free	5 U/µL	1.8 µL	1.8 µL	1.8 µL	1.8 µL
Water	Variable	Variable	Variable	Variable	Variable

**Table 4:** PCR thermocycling program temperatures and time for microorganism’s species-specific primer/probe set for each gene. The optimal annealing temperature for the species-specific primers varied between primer/probe combinations.

PCR Stage	Temp °C	Time	*E. coli*	*Salmonella*	*Aeromonas*	16S
Enzyme Activation	95	4 min	4 min	4 min	4 min	4 min
Denature DNA	95	30 s	30 s	30 s	30 s	30 s
Annealing	Varies	60 s	60 ℃	60 ℃	62 ℃	56 ℃

## Data Availability

Not applicable.
